# Mitofusins: from mitochondria to fertility

**DOI:** 10.1007/s00018-022-04386-z

**Published:** 2022-06-20

**Authors:** Shanjiang Zhao, Nuo Heng, Huan Wang, Haoyu Wang, Haobo Zhang, Jianfei Gong, Zhihui Hu, Huabin Zhu

**Affiliations:** Embryo Biotechnology and Reproduction Laboratory, Institute of Animal Science, Chinese Academy of Agricultural Sciences, Beijing, 100193 China

**Keywords:** Mitofusin, Mitochondria, Fertility, Germ cell formation, Embryonic development, Reproductive diseases

## Abstract

Germ cell formation and embryonic development require ATP synthesized by mitochondria. The dynamic system of the mitochondria, and in particular, the fusion of mitochondria, are essential for the generation of energy. Mitofusin1 and mitofusin2, the homologues of Fuzzy onions in yeast and Drosophila, are critical regulators of mitochondrial fusion in mammalian cells. Since their discovery mitofusins (Mfns) have been the source of significant interest as key influencers of mitochondrial dynamics, including membrane fusion, mitochondrial distribution, and the interaction with other organelles. Emerging evidence has revealed significant insight into the role of Mfns in germ cell formation and embryonic development, as well as the high incidence of reproductive diseases such as asthenospermia, polycystic ovary syndrome, and gestational diabetes mellitus. Here, we describe the key mechanisms of Mfns in mitochondrial dynamics, focusing particularly on the role of Mfns in the regulation of mammalian fertility, including spermatogenesis, oocyte maturation, and embryonic development. We also highlight the role of Mfns in certain diseases associated with the reproductive system and their potential as therapeutic targets.

## Introduction

Mitochondria are organelles with a double membrane structure and are capable of replication [1, 2]. As a highly complex and dynamic organelle, mitochondria are the primary site of cellular bioenergetic and lipid metabolism since they support the Krebs cycle, oxidative phosphorylation (OXPHOS), and β-oxidation [3–5]. In addition, mitochondria can change their structure and morphology by mitochondrial fusion, fission, and degradation, thus maintaining cellular function [6]. Mitochondrial fusion is essential for mitochondrial function. It has been established that the impairment of mitochondrial fusion leads to a reduction in glycolysis, OXPHOS, and the Krebs cycle [7, 8]. In parallel, mitochondria exhibit a reduction in ATP production, proton leakage, and mitochondrial membrane potential (MMP) [9]. These functional abnormalities inevitably affect cell development and eventually lead towards cellular aging and/or apoptosis.

Mitofusins (Mfns) are necessary for the maintenance of normal mitochondrial function and act by regulating mitochondrial fusion. Mfns are involved in the regulation of the structure and morphology of mitochondria at different stages of cellular development, and thus participate in the regulation of cellular differentiation, division, aging, and apoptosis [10]. In mammals, Mfns can be divided into two types, mitofusin1 (Mfn1) and mitofusin2 (Mfn2), which are widely distributed on the outer mitochondrial membrane (OMM) and are responsible for fusion of the OMM [11]. Mfn1 is located on human chromosome 3 and consists of 741 amino acids while Mfn2 is located on human chromosome 1 and consists of 757 amino acids [12]. Mitochondrial fusion and fission depend on specific genes and proteins for accurate regulation. The key gene required for fusion was first identified in Drosophila and was named Drosophila fuzzy onions protein (Fzo) [13]. Fzo is a transmembrane protein with GTPase activity that mediates the fusion of mitochondria. Since then, mitochondrial fusion proteins Mfn1 and Mfn2, homologues of FZO, were identified in mammals [14]. These proteins are key regulatory proteins for mitochondrial OMM fusion in mammalian cells [15, 16]. During cell development, Mfns directly regulate fusion of the mitochondrial membrane, spatial distribution, Ca^2+^ concentration, and the activity of OXPHOS complex subunits that are located on the mitochondrial cristae. Thus, mitochondria can respond to the energy requirements at different stages of cell development, thus maintaining cell proliferation and differentiation [17, 18].

In a manner that differs from somatic cells, spermatogenesis and oocyte maturation require two meiotic divisions and are both energy-consuming processes; these are essential for reproductive success as the ovulation of a healthy oocyte and the production of viable spermatozoa is the prerequisite to fertilization [19, 20]. As research effort intensifies in the reproductive sciences, Mfn1 and Mfn2 have been shown to be also involved in germ cell development, including spermatogenesis, follicle development, oocyte maturation, and embryo development [21–23]. In spermatogenesis, Mfns can play an indirect role in the regulation of spermatogenesis by mediating the function of Sertoli cells, and a direct role by affecting the differentiation of spermatogonia and the meiosis of spermatocytes [24]. During the development of oocytes and embryos, the expression of Mfns is precisely regulated in different stages; an excess or deficiency of Mfns leads to abnormal mitochondrial dynamics and energy metabolism, ultimately preventing oocyte meiosis and embryo development [21]. Furthermore, Mfns plays an essential role in the development and progression of several reproductive diseases in which the expression levels of Mfns are reduced [23, 25]. Gaining a better understanding of the specific mechanisms of action of Mfns in reproductive disorders could help us to identify the causes of infertility. However, thus far, few authors have attempted to review the role of Mfns in male or female reproduction, particularly with regards to their potential role in germ cell maturation and reproductive diseases. Here, we synthesize recent advances in the structural and mechanistic studies of Mfn1 and Mfn2 and their involvement in mitochondrial fusion. We focus particularly on recent progress in the role of Mfns in the regulation of spermatogenesis, oocyte maturation, and embryonic development. We also highlight the potential role of Mfns in certain diseases associated with the reproductive system and their potential as therapeutic targets.

## Structure basis of Mfns functions

**Fig. 1:**
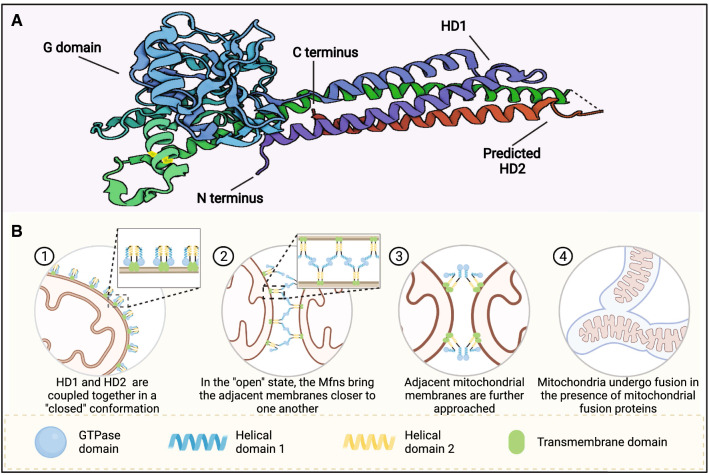
The molecular structure and mechanism of Mfns. **A** Molecular structure of human Mfns (PDB code 5GO4). **B** The fusion mechanism of the outer mitochondrial membrane

Mitochondrial fusion proteins were first reported in the 1998 [26]. With the development of crystallography, Cao et al. [27] resolved and predicted the crystal structure of Mfn1. Then, Li et al. [28] resolved the crystal structure of Mfn2 and found that Mfn1 and Mfn2 have the same topological structure; the sequence identity of both was above 80%. Mfn1 and Mfn2 share the same motifs and are composed of the GTPase domain, helical domain 1 (HD1), predicted helical domain 2 (HD2), and transmembrane (TM) (Fig. 1). Of these, the TM region is inserted into the OMM and the rest region (the GTPase domain, HD1 and HD2) are oriented towards the cytoplasm. The main role of the GTPase structural domain is to catalyze the hydrolysis of GTP to provide energy for fusion to the OMM, while HD1 and HD2 mainly pull the OMM closer, thus accelerating mitochondrial fusion. Compared to other members of the membrane fusion Dynamin family, the topology of Mfns is most similar to that of bacterial dynamin-like protein, which mediates bacterial membrane fusion [29]. The GTPase structural domain consists of eight α-helices wrapped around eight β-folds, with the two parts of HD1 and HD2 forming a four-stranded helical bundle using strong hydrophobic interactions; this is referred to as the structural domain HD1 [12, 27]. HD1 is linked to the GTPase structural domain via arginine and lysine. Mfn1 and Mfn2 can form homodimers by hydrolyzing GTP to complete fusion with the OMM. Although the sequence homology of Mfn1 and Mfn2 is high, there are significant differences in the efficiency and ability of the two to fuse membranes. Li et al. [28] found that both Mfn2 and Mfn1 form tight dimers to mediate the fusion of the OMM during the catalytic hydrolysis of GTP; however, the dimeric states of Mfn2 and Mfn1 were found to be significantly different. After the completion of GTP hydrolysis, Mfn2 remained undissociated after catalytic GTP hydrolysis; this was very different from the Mfn1 dimer that rapidly dissociated after catalytic GTP hydrolysis, thus suggesting that Mfn2 may have a stronger membrane bolus ability than Mfn1. However, Hall et al. reported that Mfn1 has a higher GTPase activity than Mfn2, and can, therefore, hydrolyze GTP faster; therefore, Mfn1 is more efficient at promoting mitochondrial fusion than Mfn2 [30]. Furthermore, Mfn2 and Mfn1 can also form heterodimers via the GTPase structural domain [28], thus suggesting that such heterodimers may play an important function in the process of mitochondrial fusion.

OMM fusion is mediated by the hydrolysis of GTP by the mitochondrial fusion proteins Mfn1 and Mfn2 [31]. Recent crystallography studies have provided a deeper insight into the molecular mechanisms underlying OMM fusion. The process of Mfn-mediated OMM fusion can be divided into three stages (Fig. 1B) [27, 28]. In the first stage, before GTP hydrolysis, the HD1 and HD2 structural domains of Mfns are coupled together in a "closed" conformation and the TM region is inserted into the OMM, with each Mfns responsible for a segment of the OMM. In the second stage, the GTPase structural domain catalyzes GTP hydrolysis, thus triggering the conformational rearrangement of the HD1-HD2 structural domain. In the "open" state, the Mfns can effectively pull the OMMs within 30 nm of one another to bring the adjacent cell membranes closer to one another. The third stage involves the formation of trans-crossover oligomers around the docking site via organized Mfns in a manner that is dependent on the hydrolysis of GTPase. The process of OMM fusion is reversible and can be controlled by the local GTP concentration and Mfns density, thus avoiding excessive fusion [27].

## Mfns in mitochondrial homeostasis

**Fig. 2:**
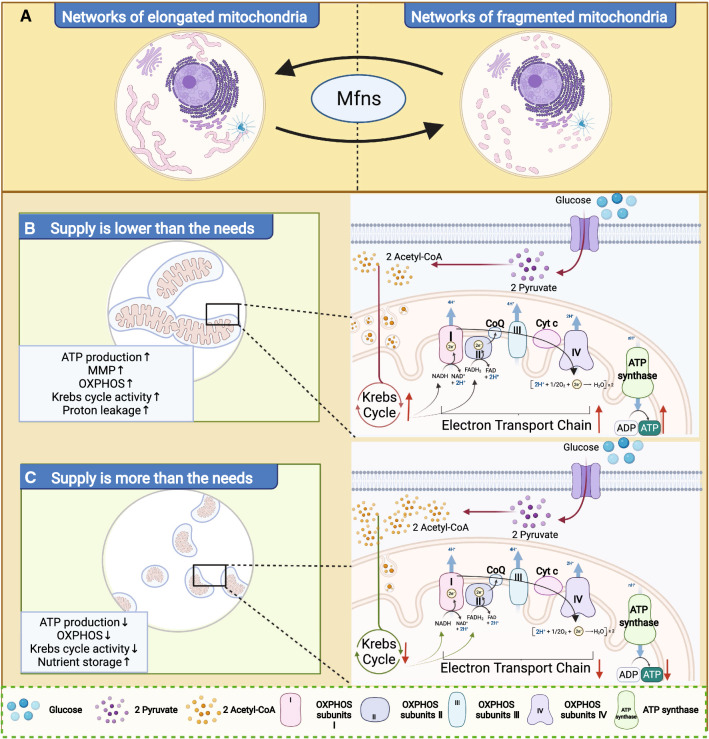
Mitochondrial morphology and cellular metabolic homeostasis. **A** The networks of elongated and fragmented mitochondrial. **B** Mitochondrial morphology and energy metabolism when supply is lower than the needs. **C** Mitochondrial morphology and energy metabolism when supply is more than the needs

Mitochondria are dynamic organelles that exhibit a highly plastic adaptation of mitochondrial morphology in response to different biological requirements and intracellular environments [32, 33]. Mitochondria undergo constant fusion and fission to form a dynamic network of mitochondrial reticulation and have two states (Fig. 2A): one is the elongation mitochondrial network, in which adjacent mitochondria undergo membrane fusion to form elongated mitochondria [34]; while the other is the fragmentation mitochondrial network, in which elongated mitochondrial membranes undergo fission and reform a network consisting of smaller mitochondria that look like fragments in the cell[35]. The purpose of morphological transformation of the mitochondrial network is to ensure that the cell can respond to various changes in energy requirements in a precise manner [15, 36].

Notably, when located in the OMM, Mfns are key proteins that regulate the morphology of the mitochondrial network [37]. Under normal physiological conditions, once the cell requires a large supply of ATP and the intracellular nutrients are not insufficient, the GTPase structural domain of Mfns catalyzes GTP hydrolysis to induce the mitochondria to convert into an elongation mitochondria network [38]. Subsequently, the network of elongated mitochondria increases the surface area between the mitochondrial membrane and the intracellular environment, thus up-regulating the efficiency of intracellular nutrient uptake (Fig. 2B). This promotes glucose oxidation, the Krebs cycle and oxidative phosphorylation, thus maintaining the MMP potential and accelerating the production of ATP, and vice versa (Fig. 2C) [9, 39]. If the cell does not require a large supply of ATP but only needs to maintain normal cellular activity, the HD1 and HD2 structural domains of Mfns will be coupled together in a 'closed' conformation to keep the common fragmented state or allowing the elongated mitochondria to convert into a network of fragmented mitochondria. Then, the efficiency of the Krebs cycle and OXPHOS would decrease; proton leakage reduces and the mitochondria produce less ATP [40, 41]. Interestingly, this fragmented mitochondrial network also acts as a more efficient nutrient storage model, especially for excess intracellular nutrients. This, may help the mitochondria to deal with a sudden increase in the demand for ATP synthesis from the organism [9].

The cristae of the mitochondria host all the complexes involved in mitochondrial respiration and are key sites for OXPHOS, including complexes I-IV, coenzyme Q, cytochrome c, and ATP polymerase [42]. In the process of OXPHOS, NADH, and FADH_2_ are re-oxidized through the respiratory chain to transfer electrons and eventually form H_2_O [43]. During electron transfer, large amounts of H^+^ are pumped out of the mitochondrial matrix to the intermembrane space, while H^+^ from the intermembrane space are returned to the mitochondrial matrix with the ATP polymerase and form large amounts of ATP [44]. Interestingly, there is a strict correlation between ATP production and the expression level of Mfns [35]. But how Mfns located in the OMM regulate the energy metabolism of the IMM is not fully understood.

Mfns are not isolated, they also convert energy metabolism (OXPHOS and aerobic glycolysis) by interacting with some specific regulatory factors, further suggesting that Mfns not only mediate mitochondrial fusion, but also directly determine cellular metabolism. The M2 isoform of pyruvate kinase (PKM2) is one of the rate-limiting enzymes in glycolysis and can promote aerobic glycolysis by forming tetramers and also switch the pyruvate of glucose metabolism from the Krebs cycle to the pentose phosphate pathway by forming dimers [45]. Li et al. showed that Mfn2 can activate PKM2 via interaction, thus promoting mitochondrial OXPHOS [46]. In response to the Mfn2-PKM2 interaction mediated OXPHOS, mTOR, a serine/threonine kinase, also participates in the Mfns-PKM2 interactions by phosphorylating Mfn2 [46]. Once phosphorylated Mfn2 interacts with PKM2, the Mfn2-PKM2 would convert the glucose metabolism pattern from aerobic glycolysis to OXPHOS to satisfy the energy requirements of specific cellular events. Apart from directly affecting the glucose metabolism pattern, Mfn2 might regulate the OXPHOS process by proton across, such as in muscle cell development, the repression of Mfn2 leads to reduced proton leakage [41]. Another study also supports this opinion in that the knockout of Mfn2 induced coenzyme Q depletion, impaired mitochondrial respiratory function, and reduced ATP production in mouse cells [47]. In these studies, the decreasing expression levels of Mfns triggered a reduction in ATP synthesis, considering that the main function of Mfns is to induce fusion of the OMM, converting the fragmentation mitochondrial network to an elongation mitochondrial network will promote ATP synthesis. Thus, the abnormal energy metabolism induced by the absence of Mfns may be due to an imbalance in mitochondrial homeostasis, where the mitochondria are unable to elongate and provide cells with a large supply of ATP.

In addition, Mfn2 regulates the expression of subunits involved in the OXPHOS complex. The depletion of Mfn2 inhibits the expression of OXPHOS I, II, III, and V subunits, thus leading to a decrease in their enzymatic activity [48, 49]. The overexpression of Mfn2 leads to an increase in the expression of several complex I, IV, and V subunits [48]. It has been shown that Mfns not only provide more energy to the cell through membrane fusion by associating with more isolated mitochondria, they also directly participate in the energy metabolism of the IMM by influencing the expression of subunits in the OXPHOS complex.

Mitochondria are not only the energy source of the cell but also an important center for the regulation of Ca^2+^, which is essential for the regulation of apoptosis and cell death [50]. Indeed, Ca^2+^ transfer from the endoplasmic reticulum (ER; one of the intracellular calcium reservoirs) to the mitochondria is required for the initiation of programmed cell death by several apoptotic factors [51]. Another function of Ca^2+^ in the mitochondria is regulated oxidative metabolism. Ca^2+^ accumulation in the mitochondria activates mitochondrial oxidative metabolism by regulating dehydrogenases and metabolite carriers [52, 53]. When mitochondrial function becomes abnormal, it will interfere with the transport of Ca^2+^ from the ER to the mitochondria [50], thus leading to an insufficient Ca^2+^ content in the mitochondria. Furthermore, the lack of Ca^2+^ support for mitochondrial oxidative metabolism leads to a secondary reduction in mitochondrial respiration and adenosine triphosphate production; this event is thought to be associated with apoptosis [54, 55]. Mfn2 plays a key role in Ca^2+^ regulation by influencing the transport of Ca^2+^ from the ER to the mitochondria [56, 57]. Mfn2 also regulates the mitochondrial response to Ca^2+^ and prevents cell fate from moving towards calcium-mediated apoptosis by converting the network morphology of the mitochondria [58]. In normal mitochondrial metabolism, Ca^2+^ transport is a fundamental event for maintaining mitochondrial homeostasis. A large number of studies have confirmed that Mfns are involved in energy metabolism and Ca^2+^ transport, thus suggesting that the normal functionality of Mfns is essential for mitochondrial homeostasis.

## Mfns in germ cell fate

Under normal physiological conditions, mitochondria can convert the state of the mitochondrial network by both fusion and fission, thus altering the efficiency of the Krebs cycle and OXPHOS to satisfying energy requirements and maintaining cell development, proliferation, and differentiation [36]. In somatic cells, as cells enter interphase, the mitochondria show an elongated network pattern and accumulate around the nucleus and cell periphery. In contrast, during most of the mitosis period, mitochondria show a fragmented network pattern that disperses in the cytoplasm [59, 60]. The development of germ cells and meiosis are far more complex than that of somatic cells. For example, both sperm and oocytes are need to undergo meiosis and complex dynamic distribution of the cytoskeleton; these processes require large amounts of ATP to maintain [61, 62].

Since Mfns regulate mitochondrial function as well as ATP synthesis, the role of Mfns in germ cells has received wide attention. The germ cells require different ATP production capacities in different stages of development [63]. The capacity of mitochondria to produce ATP mainly depends on the ability of Mfns to regulate the level of mitochondrial energy metabolism, predominantly by regulating the state of the mitochondrial network [64]. During oogenesis, there are two peak periods of ATP production, one during GVBD and the other during first polar body elimination [65]. GVBD is an important marker for the resumption of meiosis in oocytes and this process is induced by progesterone [66]. During oocyte maturation, a large majority of oocytes undergo GVBD from the germinal vesicle (GV) phase to resume the first meiotic division and eventually arrest at the metaphase of second meiosis. To satisfy the high ATP requirements of oocytes during GVBD and meiosis, Mfns can improve ATP production by regulating mitochondrial membrane fusion but can also satisfy the ATP requirement by directly regulating the activity of the OXPHOS subunits. OXPHOS is the main source of ATP production during oocyte maturation; Mfns can directly influence the supply of ATP for different phases of oocyte development by activating the subunits of OXPHOS complexes I, IV, and V, thus maintaining normal oocyte development. Once Mfns are depleted, the lack of ATP supply will inhibit chromosomal separation and oocyte meiosis as well as increasing the level of reactive oxygen species (ROS) in the cell, thus leading to autophagy and apoptosis [67].

**Fig. 3:**
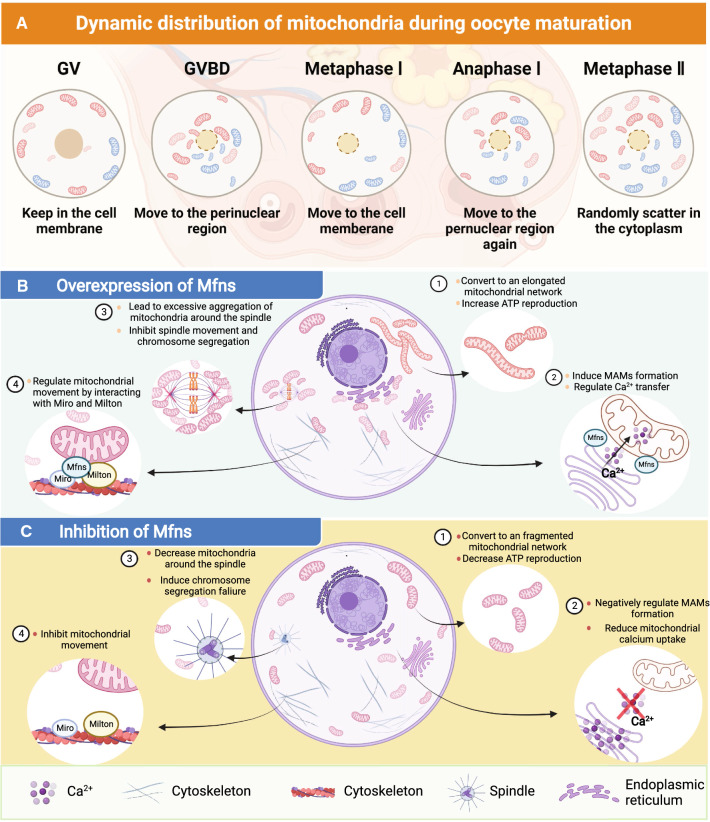
The mechanism of mitochondrial regulation of germ cell fate. **A** Dynamic distribution of mitochondria during oogenesis. **B** The effect of overexpression of Mfns on germ cell fate. **C** The effect of inhibition of Mfns on germ cell fate

Besides mitochondrial membrane fusion and OXPHOS, Mfns can also support oocyte maturation directly by regulating mitochondrial movement to determine the spatial distribution of the mitochondria. More specifically, as the oocyte develops, mitochondria gradually migrate towards the nucleus in response to GVBD and meiosis, thus exhibiting a fluctuating pattern to eventually create a uniform distribution in the cytoplasm of the mature oocyte (Fig. 3A) [68, 69]. For instance, during GV, most of the mitochondria are in the cell membrane of the oocyte, thus providing energy for intracellular protein synthesis and secretion, as well as for oocyte-granulosa cell interactions [68, 70]. After then, the mitochondria migrate towards the perinuclear region with oocytes developing from the GV to GVBD; After GVBD, mitochondria continue to diffuse toward the membrane; and mitochondria reaccumulate toward the perinuclear region at the time of polar body exclusion, this may be related to the provision of additional energy for activities such as RNA transcription [69]. Finally, mitochondria are distributed throughout the oocyte during metaphase of the second meiosis to maintain the basal metabolism of the oocyte [70]. During this process, mitochondria are anchored to the kinesin motor which is located on the cytoskeleton and subsequently transported to specific locations along the cytoskeleton, thus completing the dynamic distribution of mitochondria during oocyte development [71]. Mfn1 and Mfn2 can anchor mitochondria to the cytoskeleton by interacting with Miro and Milton proteins, thus linking mitochondria to kinesin motors. This allows mitochondria in the cytoplasm to move to specific regions [72]. This suggests that Mfns direct interactions with the microtubule-based transport apparatus. Many studies have supported this view, for instance, during oocyte maturation, the correct number of mitochondria needs to move to the spindle to ensure that the energy requirements of spindle migration are met [73]. However, following spindle migration, these mitochondria also need to be released so that they provide enough space for the spindle to be able segregate the chromosomes [74]. Previous work showed that if Mfn2 was knocked out in the oocyte, then mitochondria did not localize around the spindle [75, 76], thus suggesting that Mfn2 is involved in regulating the movement of mitochondria during meiosis. But in Mfn2-overexpressed oocytes, the release of the mitochondria surrounding spindle were absent, leading to a failure of spindle movement and chromosome segregation, ultimately cause the majority oocytes to arrest at the MI stage **(**Fig. 3B) [74, 76]. Both the overexpression and depletion of Mfn2 has been shown to affect the number of mitochondria distributed around the spindle, thus leading to meiotic failure in oocytes (Fig. 3C). These findings demonstrate that the proportion of mitochondria distributed around the spindle in meiosis can influence meiotic events.

It is evident that Mfns can regulate the dynamic distribution of mitochondria around the spindle. However, these proteins can also regulate a dynamic structural and functional link between the mitochondria and the ER, thus exerting influence on mitochondrial autophagy and cellular senescence. Gbel et al. [77] revealed that Mfn2 is also located in the ER and plays a role in regulating the formation of mitochondria-associated membranes (MAMs), which regulate the dynamic link between the mitochondria and the ER. It has also been shown that MAMs are the initiation site for autophagosome formation; this is the location of the pre-autophagosome marker ATG14 and the autophagosome-formation marker ATG5 [78]. Furthermore, MAMs are also the initiation site for mitochondrial fission, which is accomplished by wrapping mitochondria in ER tubules, and by activating ER localization proteins on MAMs, thus leading to actin multimerization and the completion of mitochondrial division [78, 79]. Another function of MAMs is to allow the exchange of Ca^2+^ between two organelles (the ER and mitochondria) [57, 80]. MAMs help to create a microregion of high Ca^2+^ within the binding region of the ER and mitochondria that can enhance the transport of Ca^2+^ to the mitochondria via mitochondrial calcium monomers [81, 82]. This Ca^2+^ uptake event via MAMs is essential for maintaining cellular bioenergetics, since this process also involves Krebs cycle regulation, dephosphorylation and the activation of pyruvate dehydrogenase [83, 84]. During the process of Ca^2+^ exchange, Mfn2 serves as an essential regulator of bound ER and the mitochondrial formation of MAMs; the depletion of Mfn2 leads to a reduction in Ca^2+^ concentration in the mitochondria and an increase in Ca^2+^ concentration in the ER [85]. This would lead to Ca^2+^ imbalance and a reduction in the Krebs cycle in the mitochondria, ultimately blocking cell development [86]. MAMs are key elements of mitochondrial involvement in cell development, apoptosis and aging, and act by regulating mitochondrial division and ER stress [87]. Not only does Mfn2 positively regulate the formation of MAMs, some studies suggest that Mfn2 can also negatively regulate the maintenance of the structure of MAMs [88, 89]. Although the specific regulatory role of Mfn2 on MAMs is not well understood, it cannot be ignored that the large distribution of Mfn2 in the structure of MAMs is involved in regulating the autophagic process (MAMs are key sites for autophagosome formation) [90]. Currently, the role of MAMs in regulating mitochondrial autophagy and Ca^2+^ transport is well established, and it has been confirmed that Mfns can regulate the function of MAMs in somatic cells; however, it is unknown as to whether Mfns can similarly regulate the function of MAMs in germ cells.

## Mfns in the development of germ cells and embryos

### Spermatogenesis and testicular development

**Fig. 4:**
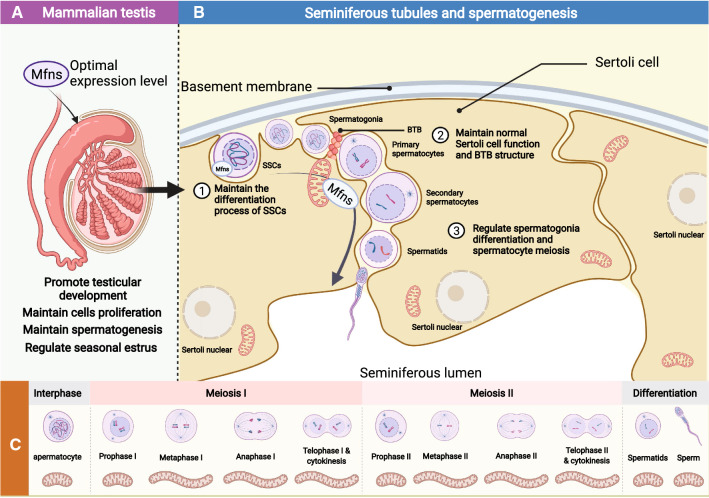
The effect of Mfns on spermatogenesis. **A** The effect of optimal expression level of Mfns on testicular development. **B** The mechanism of Mfns regulation of spermatogenesis. **C** Morphological changes of mitochondria during spermatogenesis

During spermatogenesis, the morphology, number, and position of the mitochondria in the spermatogonia, spermatocyte, and spermatozoa are constantly changing [91] (Fig. 4). In SSCs, Type A spermatogonia, and Type B spermatogonia the mitochondrial have an elliptical structure with a fragmentation network. However, as the spermatocytes gradually differentiate into round spermatids through meiosis I and II, the mitochondria are distributed around the nuclear membrane in an elongated mitochondrial network state which is regulated by Mfns (Fig. 4C). Furthermore, the number of mitochondria gradually increases, thus suggesting that the active functional state at this stage requires more ATP from the mitochondria [92]. During spermatogenesis, Mfns exhibit dynamic expression during different stages of spermatogenesis; the expression levels of Mfn1 and Mfn2 increase during the differentiation of SSCs, the differentiation of spermatogonia, and during meiosis I [22]. Mfns are important components in the regulation of mitochondrial membrane fusion and are involved in the regulation of the mitochondrial Krebs cycle and OXPHOS to maintain the ATP requirements at different stages of spermatogenesis [93]. Although their role in the maintenance of spermatogenesis is not well understood, many studies have shown that Mfns regulate the distribution and morphology of mitochondria during spermatogenesis and thus maintain the spermatogenic process.

SSCs are the basis of spermatogenesis and are located at the base of the varicocele [94]. After division of the SSCs, some of the daughter cells form new SSCs, and then complete their self-renewal [95]. The other portion of the daughter cells differentiate to form Type A spermatogonia and Type B spermatogonia [96], thus allowing meiosis. To investigate the effects of Mfns in the self-renewal and differentiation of SSCs, many studies have knocked out Mfns or inhibited the expression of Mfns [24, 97]. Interestingly, the depletion of Mfn1 and Mfn2 does not influence the self-renewal of SSCs; instead, this inhibits the differentiation of SSCs (Fig. 4B) [24]. Mfn1 and Mfn2 play a positive role in regulating the differentiation of SSCs; this maintains normal ATP production and a low level of ROS [22]. The loss of Mfn1 or Mfn2 can increase the levels of ROS that can cause an increase in DNA damage as well as inducing apoptosis [22].

SSCs undergo a series of divisions and then differentiate to form spermatocytes. The spermatocytes then undergo meiosis I and meiosis II to form round spermatozoa, which ultimately form mature spermatozoa via chromatin condensation, Golgi deformation, and the formation of the flagellum. At this stage, the mitochondria show an elongated mitochondrial network [24]. Subsequently, the round spermatids gradually transform into long spermatids and exhibit a significant up-regulation in the expression levels of Mfn1 and Mfn2 [98]. These data suggest that during sperm deformation or spermatogenesis, Mfns can convert the status of the mitochondrial network in a precise manner by mediating membrane fusion, thus providing the energy required by spermatogenesis. Furthermore, the adjacent mitochondria cluster around the nucleus and form inter-mitochondrial cement (IMC, also referred to as a pi-body, a form nuage structure). IMC contains a variety of Piwi-interacting RNA proteins and plays a role in transposon silencing, mRNA translation, and mitochondrial fusion [91, 97]. Research has identified a male germ cell specific protein, GASZ, within the IMC; it is believed that this protein is essential for the formation of the IMC and can interact with Mfns to promote mitosis in the spermatogonia and induce fusion of the adjacent mitochondria [99]. Wang et al. [97] further confirmed that nuage-associated protein GASZ could interacts with Mfn2 to regulate male germ cell development by controlling several gamete-specific mRNA fates. However, mitochondrial fusion appears to be an unnecessary function for spermatids during late spermatogenesis. Although Mfns can affect mitochondrial function by inducing mitochondria fusion and movement, the knockout of Mfn1 and Mfn2 in mice did not affect the development of spermatids (late spermatogenesis) [100]. Indeed, the level of mitochondrial fusion in spermatids is low, although sperm require the mitochondrial synthesis of ATP to maintain sperm motility and fertilization; mitochondrial metabolism in spermatids remains at low levels [100], thus indicating that after Golgi deformation, the fragmented morphology mitochondrial network morphology is sufficient to supply the spermatids with energy. Therefore, Mfns-induced mitochondrial membrane fusion might be unnecessary or not required for frequent action during late spermatogenesis.

Beside spermatogenesis, testicular development is also strongly related to mitochondrial dynamics [101]. Wang et al. [102] found that Mfns might be involved in the regulation of testicular development by regulating mitochondrial OXPHOS and the dynamic distribution of mitochondria during cell proliferation, ultimately allowing for a specific seasonal estrus in mammals (Fig. 4A). Furthermore, the testes are the source of spermatogenesis; the quality of testicular development has a direct effect on spermatogenesis and male fertility. Within the testis, the seminiferous tubules are arranged in coils and provide appropriate sites for spermatogenesis. The outer layer of the seminiferous tubules is made up of Sertoli cells, and help to maintain a normal physiological structure [103, 104]. Furthermore, the adjacent Sertoli cells are tightly connected to form a blood-testis barrier (BTB) that divides the seminiferous tubules into a basement membrane region and a lumen region. However, SSCs and undifferentiated spermatogonia are located on the outer layer of the basement membrane (near the outermost basement membrane), and as spermatogenesis proceeds, the spermatocytes need to cross the BTB to enter the lumen region of the seminiferous tubules [105]. As the germ cells cross the BTB into the lumen region, the BTB can protect them from foreign substances. In addition, the BTB can provide nutrition to the germ cells, regulate spermatogenesis through endocrine secretions, as well as organize the spermatids in the lumen. However, the effect of Mfns on the BTB during spermatogenesis has rarely been investigated. Considering the role of the BTB in spermatogenesis, future in-depth investigation of the regulatory mechanism of Mfns on the BTB will be beneficial to better understand male infertility.

In summary, Mfns can be indirectly involved in regulating spermatogenesis by mediating the functionality of Sertoli cells and can directly influence spermatogenesis by affecting the differentiation of spermatogonia and meiosis in spermatocytes. Furthermore, Mfns are involved in regulating sperm motility. In mice spermatozoa, Mfn2 is present in the flagella and co-localizes on the sperm flagella with meiosis-specific nuclear structure 1 (MNS1), which is abundantly expressed in post-meiotic sperm and necessary for the formation of normal flagella and the maintenance of sperm motility [106]. These data suggest that Mfn2 may play an essential role in maintaining the structure and function of the sperm flagella. In human spermatozoa, Fang et al. [93] found that the expression levels of Mfn2 is associated with sperm motility, but how the presence of Mfn2 in sperm maintains sperm motility is poorly understood. Based on the distribution and function of Mfn2 in human spermatozoa, it is possible that Mfn2 might promote fusion to maintain the mitochondrial structure in the sperm midpiece, and may provide ATP to sperm by regulating the levels of mitochondrial OXPHOS. By contrast, we know little about the role of Mfn1 in spermatogenesis, particularly with regards to the regulation of sperm motility. Based on research on the role of Mfn2 in maintaining spermatogonia differentiation and its involvement in regulating spermatocyte meiosis, it is possible that Mfn2 may play a more important role in the regulation of spermatogenesis.

### Follicle development and oocyte maturation

**Fig. 5:**
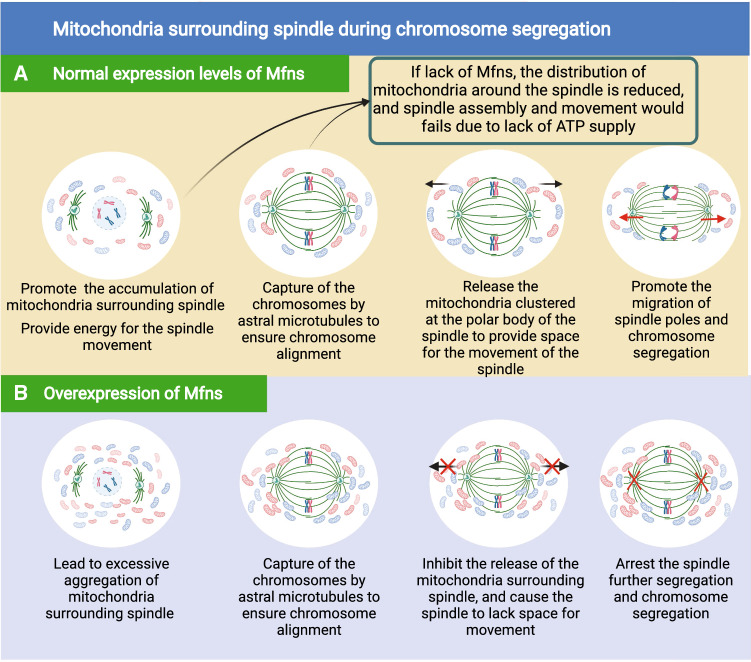
The dynamic distribution of mitochondria surrounding spindle during chromosome segregation. **A** Normal expression levels of Mfns. **B** Overexpression of Mfns

Mammalian follicles develop in the ovarian cortex, are spherical in shape, and consist of an oocyte (in the center of the follicle) and are surrounded by follicular cells [107, 108]. As the follicle develops, the oocytes within the follicle begin to mature [109]. During oocyte maturation in mammals, oocytes undergo asymmetric cell divisions and produce haploid oocytes [110, 111]. In the GV phase, the arrested oocytes resume meiosis after GVBD. Then, in MI, the spindle migrates from the central cytoplasmic region to the oocyte cortex in an actin dependent manner [112, 113]. After first polar body expulsion, the oocyte enters rapidly into the metaphase of MII and arrests at metaphase of MII until fertilization [114, 115]. Notably, these series of physiological processes are dependent on the supply of energy from the mitochondria [116, 117]. In other words, the quality of the oocyte is directly determined by the normal functionality of the mitochondria. Studies have shown that mitochondrial functionality is a key determinant of oocyte developmental potential [118, 119] and that mitochondrial dysfunction leads to meiotic defects in oocytes from obese mouse [120–123] and the arrest of pre-implantation embryos in vitro [124]. Mfns are essential for maintaining mitochondrial function and have been demonstrated to be involved in regulating follicular development and oocyte maturation in mice [125], especially during GVBD and the expulsion of the first polar body (Fig. 5A).

During follicle and oocyte development, Mfn1 and Mfn2 have similar expression levels and are involved in maintaining normal follicular development via the regulation of mitochondrial membrane fusion, mitochondrial distribution, the activity of the OXPHOS complex subunits, and spindle function [126, 127]. In mice, the abnormal aggregation of the mitochondria also disables the functionality of MAMs, thus leading to a reduction in mitochondrial OXPHOS and the Krebs cycle. In combination, the imbalance in mitochondrial homeostasis and abnormal mitochondrial distribution caused by abnormal mitochondrial function ultimately leads to meiotic failure [127, 128]. The deletion of Mfn2 in mouse oocytes leads to increased levels of ceramide; this induces apoptosis in oocytes by releasing cytochrome C from the mitochondria and activating caspases, thus triggering the arrest of follicular development [76]. Furthermore, reducing the expression levels of Mfn2 also arrest follicular development by down-regulating the mTOR signaling pathway, a serine/threonine kinase that exerts positive effects on follicle growth and development [129]. In addition to Mfn2 deletion and down-regulation, Mfn2 overexpression could affect follicular development [130]. In mice, the overexpression of Mfn2 in oocytes was shown to excessively increase the distribution of mitochondria around the spindle (Fig. 5B), this prevented spindle movement and chromosome segregation, ultimately causing most oocytes to stagnate at MI [131, 132]. The expression of Mfn2 is precisely regulated during oocyte maturation; either too much or not enough Mfn2 leads to abnormalities in mitochondrial dynamics and energy metabolism, ultimately preventing oocyte meiosis [74, 133].

While Mfn2 is essential for follicle development and oocyte maturation, it also appears that normal expression levels of Mfn1 are also necessary for follicle development and oocyte maturation. In mice, the deletion of Mfn1 can arrest the development of follicles and oocytes by down-regulating the PI3K-Akt signaling pathway [134]; this is the basis of oogenesis and acts by positively regulating oocyte–granulosa cell interactions to promote oocyte development [135]. In addition, Hou et al. [19] showed that the knockout of Mfn1 in mice reduced the number of follicles, and most follicles were arrested at 5–8 weeks with an abnormal distribution of mitochondria in oocytes.

These studies showed that Mfn1 and Mfn2 play a key role in maintaining follicle development and oocyte maturation. However, some studies also reported that Mfn2 might not affect follicle development and oocyte maturation [19, 136]. For instance, the specific knockout of Mfn2 in oocytes has no effect on ovulation and parturition in mice [19]. The oocyte-specific deletion of Mfn1 (but not Mfn2) results in an interruption in the communication between oocytes and somatic cells, and leads to impaired follicular development at the preantral-to-antral follicle transition [136]. Unlike Mfn2, current research findings indicate that Mfn1 is positively involved in the regulation of follicle and oocyte development. Data suggest that Mfn1 might play a more important role in maintaining follicle and oocyte development than Mfn2. However, it remains unclear exactly why Mfn1 and Mfn2 are not similarly effective in regulating oogenesis, although Mfn1 and Mfn2 share the same topology and motifs and have 80% sequence identity. It is possible that the sequence differences between Mfn1 and Mfn2, or the higher structure of the proteins encoded by these genes, might influence their functionality in follicle and oocyte maturation.

### Embryonic development

After maturation of the mammalian oocyte, the sperm and oocyte meet at a specific location in the oviduct of the female animal. The male and female gametes then fuse to form a zygote that includes all the genetic material required for the development a new individual [137, 138]. Subsequently, the zygote develops into an individual in a suitable maternal environment. The zygote divides and undergoes densification, eventually forming a blastocyst which migrates to the uterine horn. Blastocysts consist of an endocyst and trophectoderm, which continue to differentiate to form the ectoderm, mesoderm, and endoderm. Next, the ectoderm plays a role in the formation of the nervous system and epidermis, the mesoderm develops mainly into connective tissue and the circulatory system, and the endoderm and trophectoderm play roles in the formation of the placenta [139].

**Fig. 6:**
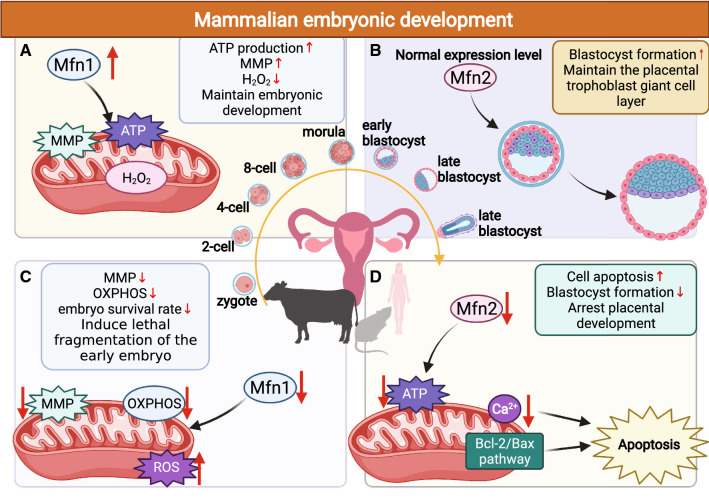
The effect of Mfns on mammalian embryonic development. **A** The effect of up-regulating Mfn1 expression levels on mammalian embryo development. **B** The effect of normal expression levels of Mfn2 on mammalian embryo development. **C** The effect of down-regulating Mfn1 expression levels on mammalian embryo development. **D** The effect of down-regulating Mfn2 expression levels on mammalian embryo development

Early embryo development and implantation are complex and energy-consuming events, with the embryo forming in the oviductal jugular and then migrating through the oviduct, passing through the 2-cell, 8-cell, and blastocyst stages, and finally moving to the uterus (Fig. 6) [140, 141]. Mfns are necessary for embryo development, and participate by regulating mitochondrial fusion and homeostasis, thus maintaining ATP and MMP at normal levels during embryo development [142]. Hua et al. [143] previously demonstrated that high levels of Mfn1 expression increased mitochondrial ATP synthesis, MMP levels, and decreased the levels of H_2_O_2_ in bovine somatic cell nuclear transfer (SCNT) embryos, which is useful for development in early SCNT embryos (Fig. 6A). Instead, during early embryonic development, the low expression levels of Mfn1 led to the lethal fragmentation of the early embryo by disrupting mitochondrial MMP and OXPHOS components, ultimately resulting in a reduction in embryo survival rate (Fig. 6C) [144]. These data indicate that maintaining Mfn1 at certain levels is necessary for embryonic development. Similarly, the normal expression of Mfn2 maintains blastocyst formation (Fig. 6B), although once the expression of Mfn2 decreases, it triggers mitochondrial dysfunction and induces apoptosis through the Bcl-2/Bax and Ca^2+^ pathways, ultimately reducing the rate of blastocyst formation and the speed of cleavage the mouse uterus (Fig. 6D) [145, 146]. Furthermore, the lack of trophoblast giant cells in Mfn2 mutant mice leads to placental development arrest and death during mid-gestation; this is attributed to reduced mitochondrial fusion and ATP supply during embryogenesis due to Mfn2 deficiency [26]. Therefore, Mfns might be an important regulator to support embryonic development. However, how Mfns are involved in regulating the process from zygote to early embryonic development and embryonic implantation is poorly understood. It is worth considering whether Mfns are associated with embryo-induced infertility, such as early embryonic loss in humans as well as animal models, such as the bovine and porcine model.

## Mfns in reproductive diseases

**Fig. 7:**
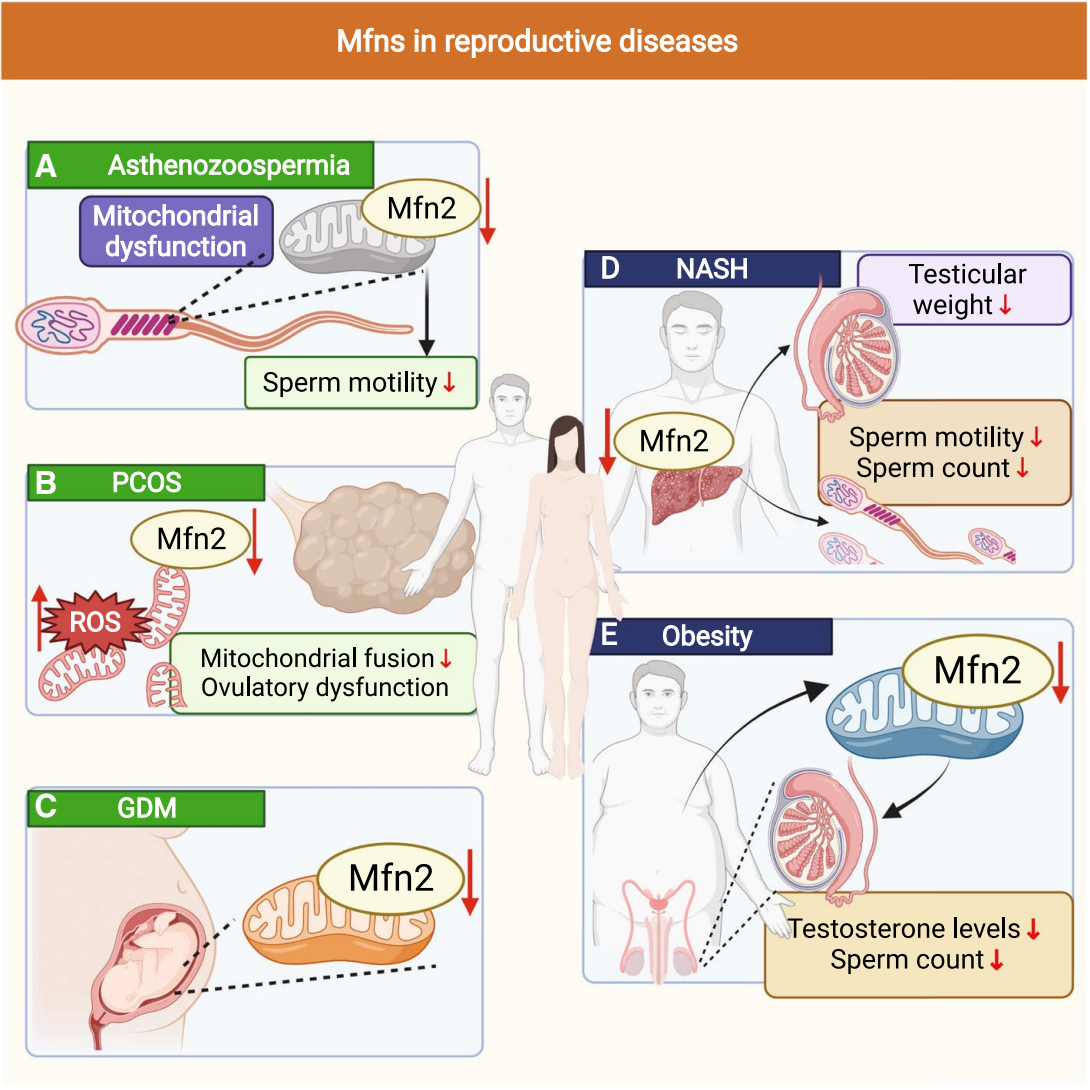
Effect of Mfn2 on the development of some reproductive diseases and some certain nutritional and metabolic diseases. **A** Effect of Mfn2 on asthenozoospermia. **B** Effect of Mfn2 on polycystic ovary syndrome (PCOS). **C** Effect of Mfn2 on gestational diabetes mellitus (GDM). **D** Effect of Mfn2 on non-alcoholic steatohepatitis (NASH). **E** Effect of Mfn2 on obesity

Mfns serve as key proteins for the induction of mitochondrial fusion that are important for mitochondrial dynamics as well as mitochondrial function and homeostasis [147]. Therefore, the onset and development of many diseases are closely associated with Mfns, such as cancer, neurological diseases, obesity, and vascular diseases [148–152]. Interestingly, recent discoveries have revealed that several reproductive diseases are also closely associated with Mfns, such as asthenozoospermia, polycystic ovary syndrome (PCOS), and gestational diabetes mellitus (GDM) (Fig. 7). Asthenozoospermia is characterized by a progressive decrease in sperm motility and is frequently seen in infertile men with primary ciliary dyskinesia or immotile ciliary syndrome [153, 154]. In asthenozoospermic patient, the sperm exhibits mitochondrial dysfunction with a markedly reduced expression level of Mfn2 (Fig. 7A); this may be the trigger for the reduced sperm motility seen in asthenozoospermia and may arise by the regulation of mitochondrial dysfunction [93]. This suggests that the specific regulation of Mfn2 expression levels in the sperm of patients might help to alleviate asthenozoospermia and the reduction in progressive sperm motility caused by mitochondrial dysfunction. In females, PCOS is one of the most common diseases that affect women with abnormal endocrine and metabolic conditions that are characterized by ovulatory dysfunction and hyperandrogenemia; the main characteristics are an irregular menstrual cycle and infertility [155, 156]. Previous research in PCOS mice showed that mitochondrial fusion/division was disturbed, the levels of ROS were increased, Mfn2 expression was significantly reduced (Fig. 7B) [25]. Selenium treatment led to an improvement in the abnormal expression of Mfn2 and successfully ameliorated PCOS-related changes in the metabolic phenotype, thus suggesting that Mfn2 may play an essential role in the pathogenesis of PCOS. In addition, the expression levels of Mfn2 were significantly reduced in the women placenta with GDM, but whether Mfn2 is a trigger for GDM pathogenesis and the role of Mfn2 in the induction of GDM are not well understood (Fig. 7C) [157].

Except for asthenozoospermia, PCOS and GDM, Mfns can also induce certain nutritional and metabolic diseases, such as non-alcoholic steatohepatitis (NASH) and obesity. NASH is characterized by the excessive accumulation of lipids in the liver, which can lead to liver fibrosis, cirrhosis, and hepatocellular carcinoma. Mice with NASH are known to exhibit a reduction in testicular weight, sperm count, and sperm motility (Fig. 7D) [158]. The expression levels of Mfn2 in the liver are known to be closely related to the development and progression of NASH [159, 160]. Mice with NASH show a significant reduction in Mfn2; in addition, the re-expression of Mfn2 improved the disease, thus suggesting that regulating the expression levels of Mfn2 in the liver of NASH might potentially serve as a therapeutic target for male reproductive function [159]. Like NASH, obesity has also become a major public health problem and can lead to central dysregulation of the hypothalamic-pituitary–gonadal axis, thus inducing diseases of the reproductive system. In females, obesity-induced reproductive diseases include menstrual irregularities, pregnancy complications, and infertility (caused by anovulation). In males, obesity-induced reproductive diseases can lead to infertility due lower testosterone levels and a reduced sperm count (Fig. 7E) [161]. Strikingly, humans with obesity exhibit a reduction in the expression levels in Mfn2. In mice, the specific knockout of Mfn2 in adipocytes resulted in increased food intake, impaired glucose metabolism, and increased adiposity [162, 163]. This suggests that improving the expression levels of Mfn2 in obese patients may help to treat male or female infertility caused by obesity. The abnormal expression of Mfns not only affects the reproductive success of the parents, but also has an impact on the health of the offspring. Chiaratti et al. [126] previously showed that the oocyte-specific deletion of Mfn2 caused weight gain and glucose intolerance in the offspring; these findings were consistent with those of Garcia et al. [164] who reported that phenotypic abnormality (weight gain and glucose intolerance) in the offspring might relate to the disruption of functionality of MAMs in oocytes following the oocyte-specific deletion of Mfn2.

As the functions of Mfns in spermatogenesis, oocyte maturation and early embryonic development are gradually revealed, Mfns are also emerging as potential targets for the therapy of reproduction-related diseases. For instance, recent research involving the treatment of t testicular damage induced by the anticancer drug doxorubicin showed that the injection of doxorubicin into rats resulted in reduced expression levels of Mfn2 in the testis; this induced the disruption of mitochondrial fusion [165]. The administration of alfa lipoic acid to rats exposed to doxorubicin was shown to improve the expression levels of Mfn2, and thereby alleviate doxorubicin-induced testicular injury [165]. Another study, relating to testicular damage caused by Cadmium (Cd) also found that an intraperitoneal injection of Cd into rats resulted in reproductive toxicity, including poor semen quality, male infertility, and reduced expression levels of mRNA for both Mfn1 and Mfn2 [166]. However, the intraperitoneal injection of vitamin E improved the mRNA expression levels of Mfn1 and Mfn2, thus improving Cd-induced poor semen quality and male infertility [166]. Thereby, Mfns is not only associated with reproductive diseases by triggering mitochondrial dysfunction, these proteins are also involved in various reproduction-related diseases and might represent potential therapeutic targets for the treatment of reproduction diseases.

## Conclusions

Mfns maintain the bioenergetics required for all stages of germ cell formation and embryonic development and act by regulating mitochondrial network morphology, mitochondrial movement, Ca^2+^ homeostasis, the subunits of OXPHOS complex activity and the connections with other organelles. The main feature in the past few years was that Mfns were reported widely in different reproductive system model, providing more novel mechanism determining male or female fertility. However, there are still several key challenges to overcome to making Mfns of critical interest for potential new therapeutic approaches. For instance, as “nurturing cells”, it will be interesting to understand the Mfns functions in the Sertoli cells and their regulatory roles in maintaining the blood-testis barrier and the SSCs homeostasis of self-renew and differentiation, which in turn impact the continuous production of sperm and male fertility. Meanwhile, mitochondrial fusion is also essential for steroid production in Leydig cells [167], so Mfns regulate steroidogenesis in Leydig cells also merit further investigations. Other interest aspect is that the role of Mfn2 and Mfn1 in folliculogenesis and spermatogenesis is different. Mfn1, but not Mfn2, is essential for oocyte development and folliculogenesis [19]; whereas Mfn2 plays a more prominent role in regulating spermatogenesis [97]. It indicates that Mfn2 and Mfn1 have different regulatory roles in the process of folliculogenesis and spermatogenesis, and further studies are required to explore the different underlying molecular mechanisms of MFN1 and MFN2 in the regulation of germ cell development.

Additionally, although current research related to Mfn2 and reproduction has provided us with a wealth of therapeutic ideas for alleviating or treating early embryo loss and death, PCOS, asthenospermia, and other common human reproductive disorders. However, the role of Mfn2, especially the upstream and downstream molecular mechanisms mediating the effects of Mfn2 on reproductive diseases has yet to be dissected; in particular, studies that specifically target the reproductive disorders caused by Mfn1 have yet to emerge. Therefore, there is still much scope to explore the role of Mfns in mammalian reproduction, which made Mfns of particularly crucial interest for potential new therapeutic approaches.

## Data Availability

Not applicable.
